# The impact of nature video exposure on pro-environmental behavior: An experimental investigation

**DOI:** 10.1371/journal.pone.0275806

**Published:** 2022-11-08

**Authors:** Lisette Ibanez, Sébastien Roussel

**Affiliations:** 1 CEE-M, University Montpellier, CNRS, INRAE, Institut Agro, Montpellier, France; 2 CEE-M, Univ. Montpellier, CNRS, INRAE, Institut Agro, University Paul Valéry Montpellier 3, Montpellier, France; 3 EPSYLON, University Paul Valéry Montpellier 3, Montpellier, France; Yunnan University of Finance and Economics, CHINA

## Abstract

We analyze whether exposure to a nature documentary increases pro-environmental behavior (PEB). We test this causal link in an experiment where subjects viewed a video featuring either an urban (control treatment) or a nature setting (nature treatment). We consider two types of behavior: a monetary donation to an environmental non-governmental organization (ENGO) that we call an eco-donation, and subsequently, a non-monetary decision (i.e., recycle or not recycle headphone protectors) that we call an eco-action. We find that virtual exposure to nature boosts both eco-donation and eco-action. Interestingly, the increase in PEB only occurs for individuals who express low environmental values. We did not find any negative or positive spillover effects on the eco-action. We finally provide robustness checks and discuss policy implications.

## Introduction

Pro-environmental behavior (PEB) includes various activities such as limiting energy consumption, avoiding waste, recycling, and voting for green political parties [1]. The main barrier that prevents individuals from achieving PEB is that such behaviors are costly [2–4]. For example, switching to organic food incurs a monetary cost [5], or selective sorting and recycling require time and thus incur opportunity costs [6–7].

Behavioral-based interventions to improve PEB may be unrelated to monetary means, such as nudges which are non-binding incentives that could guide individuals’ decisions [8,9], e.g., through social norms [10,11] and emotional states [12,13]. Another way to promote PEB through behavioral-based interventions may rely on enhancing people’s exposure to nature. Indeed, as individuals would logically be more inclined to protect the environment if they felt they belonged to larger ecosystems [14,15], reminding people of the natural world in their daily surroundings or workplaces could be an effective and low-cost strategy. Individuals spend a large part of their time indoors and away from nature because of their jobs and personal activities [16,17], and more broadly urbanization leads to a lack of ecological awareness [18], which contributes to a physical and psychological disconnect. Hence, exploring the impact of exposure to nature, especially by relying on virtual environments, could affect PEB significantly.

Exposure to nature favoring connectedness has repeatedly been proven to have positive effects on individuals linked to biophilia. Recent contributions in the health studies literature have tried to better understand these benefits including increased physiological well-being through stress reduction and lower blood pressure [19,20] and increased psychological well-being through cognitive performance and positive emotions [20–22], impulsivity control [23], attention restoration [24], mindfulness [25], and mental health more broadly [26]. Another strand of literature in conservation biology focuses on connection and life experiences with nature related to environmental concerns, especially towards biodiversity [27], and stresses that exposure to nature is linked to PEB [28]. Whitburn et al. [28] as well as Barragan-Jason et al. [29] conduct meta-analyses and highlight that a stronger connection to nature may then result in greater engagement in PEB. In environmental psychology and communication studies, various contributions stress that exposure to nature is significantly associated with PEB. They identify the causality between stated person–environment relationships and PEB [30], between virtual exposure to nature through videos and cooperative sustainable behaviors regarding natural resource management [31], and between virtual exposure to nature or biodiversity conservation videos and charitable giving [32,33]. More precisely, Zelenski et al. [31] use laboratory experiments to test the effects of viewing brief videos containing natural versus built spaces, and analyze whether nature produced more cooperative and sustainable behavior by using an iterative monetary-incentivized fishing game to reflect a commons dilemma. They show that virtual exposure to nature (videos) not only generates cooperative behavior but also induces higher social value orientation scores and sustainability intentions. Arendt and Matthes [32] in their laboratory experiment implement two treatments where participants either watch a nature documentary or a documentary about Einstein’s theory of relativity (control). Their study focuses on connectedness to nature and whether this connectedness results into PEB. PEB consists in a €1 donation to one of eight causes; two nature-related causes compared to six “control” causes. They show that a mediated nature experience is not sufficient to elicit an increase in connectedness to nature whereas exposure to a nature documentary influences PEB positively, i.e., more individuals donate money to nature-related organizations compared to nature-unrelated organizations after having watched the nature documentary. Finally, Shreedhar and Mourato [33] also use a laboratory experiment with different treatments. Each participant watches one nature-related video, featuring either a non-charismatic species (Bats), a charismatic species (Lions), or a composite habitat (Savanna) composed of both species, with and without additional content on human impact. The aim of their study is to analyze whether charismatic species, habitats and information about human-caused endangerment drive charitable giving; they also control for public recognition and past prosocial behaviors. They highlight that charitable donation is responsive to the mediated content watched with distinct effects on the probability of donating and the amount donated. Specifically, videos depicting charismatic Lions increase the probability of donating compared to uncharismatic Bats though do not increase the amount donated, whereas videos on human-caused endangerment increase the amount donated (conditional on deciding to donate).

Nevertheless, previous contributions either do not measure PEB in a fully consequential manner as this is stressed by Klein and Hilbig [18], or only deal with one specific PEB. More precisely, Zelenski et al. [31] measure PEB with a behavioral intention scale and thus reflects a behavioral intention which cannot be considered as a full consequential PEB. On their side, the PEB measure of Arendt and Matthes [32] only studies the likelihood of individuals donating money to nature-related organizations versus nature-unrelated organizations as a control. In other words, participants necessarily donate €1 and only choose the recipient, which underestimates the consequentiality and the range of PEB. Furthermore, Shreedhar and Mourato [33] offer a broader range of donations as a part of an adapted dictator game to an environmental organization. However, they focus on biodiversity with solely nature-related videos whilst framing for charismatic species, habitat, and human impacts features. In addition, the selection of the environmental organization as a recipient is unique and linked to biodiversity with emblematic species.

Klein and Hilbig [18] criticize the previous measures arguing that these are either confounded with cooperation or not fully consequential. They investigate PEB as a consequential measure through two experiments whilst using the greater good game [34], which is an adaptation of the public good game separating contributions by individuals into to three accounts, i.e., a private account, a shared account common for individuals within the same group (cooperation between players in anonymous groups of three individuals), and an environmental account corresponding to an adapted dictator game in donating to an environmental organization. In those two experiments, participants were randomly assigned to different treatments: a nature video, a video with social interaction, or a video of an urban environment as a control (experiment 1); an intact nature video, a destroyed nature video, or a video of an urban environment as a control (experiment 2). They show that increasing the salience of nature per se does not increase PEB. They also show that in contrast increasing the salience of the relevance of PEB towards nature exposure could increase PEB, although at the cost of cooperation. So, their approach does assess a consequential measure in the sense of its impact on the environment. However, they only consider monetary donation as the sole PEB, and do not analyze the impact of nature exposure on consecutive sustainable behaviors.

Kollmuss and Agyeman [2] distinguish PEB between direct and indirect environmental actions, which do not have the same type of implications for people and that may interact with each other. In other words, donating to an environmental organization or green voting corresponds to an indirect PEB, whereas reducing energy consumption or recycling corresponds to a direct PEB, questioning potential spillover issues on later subsequent behavior [35]. As stressed by Ghesla et al. [35], spillover effects are identified in the literature from a first decision to a second decision where the first decision is different from the second one, leading either to positive, negative, or no spillover. For example, Lanzini and Thøgersen [36] show the existence of positive spillovers in order to avoid cognitive dissonance, i.e., people who bought eco-labeled product tend to adopt more other pro-environmental actions, such as turning off the light when leaving a room. However, spillover effects can go in the opposite direction. For instance, Clot et al. [37] show through a laboratory experiment that when participants perform PEBs in a previous stage, their willingness to donate to an environmental charity reduces. Or, Tiefenbeck et al. [38] demonstrate that households who reduce their water consumption simultaneously increase their electricity usage. This means that environmental policies to promote sustainable behavior can have counterproductive side effects and wreck initial benefits. To anticipate such eventual side-effects, a better knowledge on spillover effects is needed.

Consequently, in this paper, we analyze whether virtual exposure to nature affects two distinct types of PEBs and whether spillover effects appear when considering these two PEBs. We investigate this question using a laboratory experiment in which participants are virtually exposed to either of two distinct surroundings: a nature surrounding and an urban surrounding. We consider two types of PEBs. First, we examine a monetary donation to an ENGO that we call an eco-donation (indirect PEB). Second, we investigate a recycling action through a green deed that we call an eco-action (direct PEB). This allows us to explore observed behaviors and to analyze whether virtual exposure to nature affects PEB in two ways: whether exposure to a virtual natural environment affects these two distinct types of PEBs and whether spillover effects appear (more specifically, whether a monetary decision reinforces (or not) a non-monetary decision). We also elicit ecological awareness of participants via the New Ecological Paradigm (NEP) scale [39,40] in order to link subjects’ stated environmental values [41] to their real PEB; note that we indifferently use the terms environmental values and beliefs in the remainder of the paper following Aguilar-Luzón et al. [41].

With respect to our overall literature review, we investigate how exposure to nature can increase PEB and more deeply our motivations can be stated as follows. First, our aim is to extend the literature by considering two different types of PEBs, namely, an indirect one (monetary donation) and a direct one (recycling) [2]. Second, our aim is to make an experimental contribution based on our approach of using virtual exposure to nature, which extends previous contributions by measuring consequential behavior fully [18,31,33]. Linked to these both motivations, our purpose is to test the existence of spillover effects on subsequent PEB [35] and to assess consistency or inconsistency between direct and indirect PEB [37,42]. Third, with regards to ecological awareness, our aim is to relate the potential impact on PEB to individuals’ environmental beliefs [41].

Thus, we operationalize these motivations in investigating three behavioral hypotheses. Our first hypothesis is linked to the potential impact of nature manipulation: being exposed to a virtual natural environment encourages individuals to act pro-environmentally by increasing both eco-donations and eco-actions [2,18,32,33].

### Hypothesis 1

Being exposed to nature compared with an urban environment through video footage positively impacts PEB.

Our second hypothesis emphasizes a potential spillover effect [35]. This hypothesis complements Hypothesis 1 by analyzing the interplay between both PEBs by investigating whether the impact on eco-donations of exposure to nature affects eco-actions. This allows us to test for consistent or inconsistent behavior [37,42], i.e., if people reinforce or not their pro-environmental actions for the two types of PEBs at stake.

### Hypothesis 2

Being exposed to nature compared with an urban environment through video footage impacts PEB through a spillover effect: exposure to nature impacts eco-donations, which in turn impacts eco-actions.

Further, we examine if subjects’ stated environmental values, measured by the NEP scale, influence their PEB. So, our third hypothesis supplements the previous hypotheses. We suppose that individuals with a high degree of environmental beliefs behave in a more environmentally friendly way than individuals with a low degree of environmental beliefs. Indeed, Aguilar-Luzón et al.’s [41] literature review shows empirical evidence that people who adhere more strongly to ecocentric beliefs act in favor of the environment. Consequently, investigating the impact of environmental preferences, we suppose that exposure to nature impacts PEB according to the individual’s degree of environmental beliefs. To the best of our knowledge, the literature has not yet explained why virtual exposure to nature works (or does not work) in different populations based on their environmental beliefs.

### Hypothesis 3

Being exposed to nature compared with an urban environment through video footage positively impacts PEB to a greater extent for those with a high degree of environmental beliefs.

The remainder of this paper is organized as follows. In Section 2, we present our experimental protocol. In Section 3, we present our results. In Section 4, we provide a robustness analysis of our results. Finally, we offer a discussion and concluding remarks in Section 5.

## Materials and methods

### Experimental design

Our research strategy and experimental design are structured as follows. First, we measure the stated environmental beliefs of participants using the new ecological paradigm (NEP) scale [39,40]. This scale, based on stated preferences, is valid and reliable, as it correlates highly with real PEB [43]. More precisely, we use the NEP scale to measure an individual’s degree of endorsement (from low to high) of an ecological worldview using a 15-item survey with the scores for each item ranging on a Likert-type scale between 1 and 5. We use the French version of the NEP scale [44]. Respondents are asked to indicate the extent to which they agree with these 15 items. The answers are then used to develop various statistical measures of environmental values either by grouping the items into five three-item categories focused on the *limits to growth*, *anti-anthropocentrism*, *balance of nature*, *anti-exemptionalism*, and the current perception of a *major ecological crisis* or by grouping all the items to obtain overall and average results. The higher the score, the greater one values the environment.

Second, participants watch a 12-minute video on his/her computer terminal; both videos and associated text edits are available in S1 File. We designed the experiment in a similar way as Zelenski et al. [31] and Klein and Hilbig [18]: participants are exposed either to nature or urban stimuli, and the urban environment video is the benchmark. The argument put forward is: “that nearly every part of the video contains human-built spaces (which) makes it antithetical to common conceptions of nature [45]. Thus, these excerpts were relatively ‘pure’ representations of nature and non-nature.” [31]. We randomly assign participants to one of the two treatments (between-subjects design). In Treatment T1 (“urban”), participants view an excerpt from *Walks with an architect series* by Landmark Media Inc. This is a documentary on New York City architecture, which shows images of the city and plans for building construction. A voiceover explains how, when, and why the tallest buildings, mainly on Broadway Avenue, were built. In Treatment T2 (“nature”), participants view an excerpt from *Wild Yellowstone* by the National Geographic Channel. This is a documentary on Yellowstone Park made up of images of the park’s fauna and flora. The audio includes melodies and a voiceover describing the interactions between species. The voice off excludes any moral or pro-environmental message in order to prevent priming effects. In the literature, video is a conventional means of exposing individuals to natural settings and investigating their connectedness to nature and PEB [32,46]. Moreover, nature videos as a virtual means can be directly linked to biodiversity conservation issues and charitable giving [33]. To avoid any priming effects, the nature video excludes “moral” and pro-environmental messages to prevent a clear link between video exposure and following experimental tasks; moreover, our between-subjects design excludes a comparison of the video content across conditions. We also assess the emotional impact of participants watching the video using the affective slider scale [47] by measuring their pleasure and arousal before and after, as in particular the pleasure dimension is strongly correlated with the pairs of words unhappy/happy, annoyed/pleased, and unsatisfied/satisfied identified in the self-assessment manikin scale used to measure emotion [48]. The affective slider scale is a continuous measurement scale that uses a cursor to be moved on a horizontal axis. On the far left of this axis appears a sad face emoji, while on the far right appears a happy face emoji. The individual must move the cursor left or right to express his or her emotional state. This axis is implicitly limited from 0 to 100 without this number being visible to participants. This scale is used for the measurement of pleasure and emotional arousal. While watching the video, participants are provided with an audio headset with headphone protectors in the form of hygienic coverings.

With regards to eco-donations, we rely on the dictator game [49,50]. The dictator game is interesting for testing generosity and prosocial behavior because there are no strategic interactions between players. Indeed, the dictator can transfer money to the recipient who is not able to refuse or return this money. Donation behavior can be explained by the expected warm-glow [51] and identity of the recipient [52]. Eckel and Grossman [52] indeed show through the American Red Cross example that donations are higher when the recipient is an NGO rather than an individual. In our study, we invite participants to play a modified dictator game in which the recipient is an ENGO. Consequently, we assess PEB and expect greater donations than in a standard dictator game [12,13]. Each participant is given a €10 endowment and must indicate the amount (an integer between €0 and €10) to donate to the ENGO (this amount was actually paid at the end of the experiment). The experiment is then incentivized with a split between the part of the endowment kept for private use and amount donated. To avoid any anchoring effect and cover international, national, and local actions, participants can choose among four ENGOs: the World Wildlife Fund (the world’s leading nature conservation organization), *Fondation pour la Nature et l’Homme* (a French non-political organization), *France Nature Environnement* (French Federation of Organizations for the Protection of Nature and the Environment), and *Ouvre-Tête Alternative Sociale et Solidarité Écologique* (a student union promoting sustainability created in 2006 at the University of Montpellier).

With regards to eco-actions, we design a real green deed in our experiment. We offer participants the possibility of making a non-monetary decision, namely, recycling a disposable good in a dedicated recycle bin. As stated above, we provide participants with an audio headset with hygienic headphone coverings at their arrival and invite them during the instructions to dispose these coverings in the recycling bin made available at the end of the experiment in the payment room.

### Experimental procedure and subject pool

The experiment was conducted at the Laboratory for Experimental Economics in Montpellier (LEEM) in sessions run between December 5 and December 18, 2017. In total, 113 subjects were recruited randomly from the LEEM database following the ORSEE software procedure [53], if they had not previously participated in any dictator game-type experiment. The single-blind experiment was computed using the Python programming framework for experimental economics.

The whole procedure was reviewed and approved by the CEE-M Institutional Review Board (Univ. Montpellier–CNRS–INRAE–Institut Agro). The full ethics statement is set up as follows: “*The present project has been assessed according to the different articles of the CEE-M’s Ethical Charter (approved by the CEE-M’s scientific council)*, *namely*: *dignity and integrity (A1)*, *information and consent (A2)*, *confidentiality and data protection (A3)*, *storage and availability of the protocols and data (A4)*, *scientific integrity (A5)*. *Based on the referees’ report*, *the Review Board considers that this project perfectly follows the rules established in the Ethical Charter*.”

Subjects were informed that the experiment was on economic decision-making. The maximum number of subjects per session was 20. The experimental procedure was as follows. We randomly assigned an experimental treatment to each session. Within each session, each subject was randomly assigned to a booth with a computer terminal, consent was informed and subjects provided written consent to participate. Subjects did not have any interaction with each other.

The successive steps were then as follows (see the instructions in S1 File): first, we asked subjects to rate their level of pleasure and arousal upon arrival; second, we asked them to choose an ENGO with which they wanted to be associated for the duration of the experiment to avoid any latency effect between the exposure and eco-donation decision; third, we asked them to rate the statements constituting the NEP scale; fourth, subjects watched one of the urban or nature videos and then rated their level of pleasure and arousal again; fifth, they made their eco-donation decision through the adapted dictator game; and sixth, they answered sociodemographic questions (see the sociodemographic questions in S2 File). At the end of the experiment, subjects were invited one at a time to enter the payment room to collect their earnings privately. During the payment process, subjects had the opportunity to make the eco-action by throwing the headphone coverings in the dedicated recycling bin. According to each participant’s decision, we divided the initial endowment (€10) into two envelopes: one with the individual’s earnings (including the show-up fee) and the other with the individual’s contribution to the chosen ENGO. We asked participants to check that the amounts in the two envelopes were accurate and we assured them that the monetary donations would be sent to the selected ENGO. Average earnings, including the show-up fee, was €12. The experiment lasted about one hour.

Out of the 113 participants, 56 subjects are assigned to the urban treatment (T1) and 57 to the nature treatment (T2). Subjects are similar in age (T1: *M*_*Age*_ *=* 22.79 years, *SD =* 4.13; T2: *M*_*Age*_ = 22.74 years, *SD =* 4.13). There is an overrepresentation of men in the urban treatment (T1) (64.3%) compared with the nature treatment (T2) (49.1%). Most subjects are full-time students (T1: 83.9%; T2: 93%).

## Results

### Descriptive analysis

**Fig 1:**
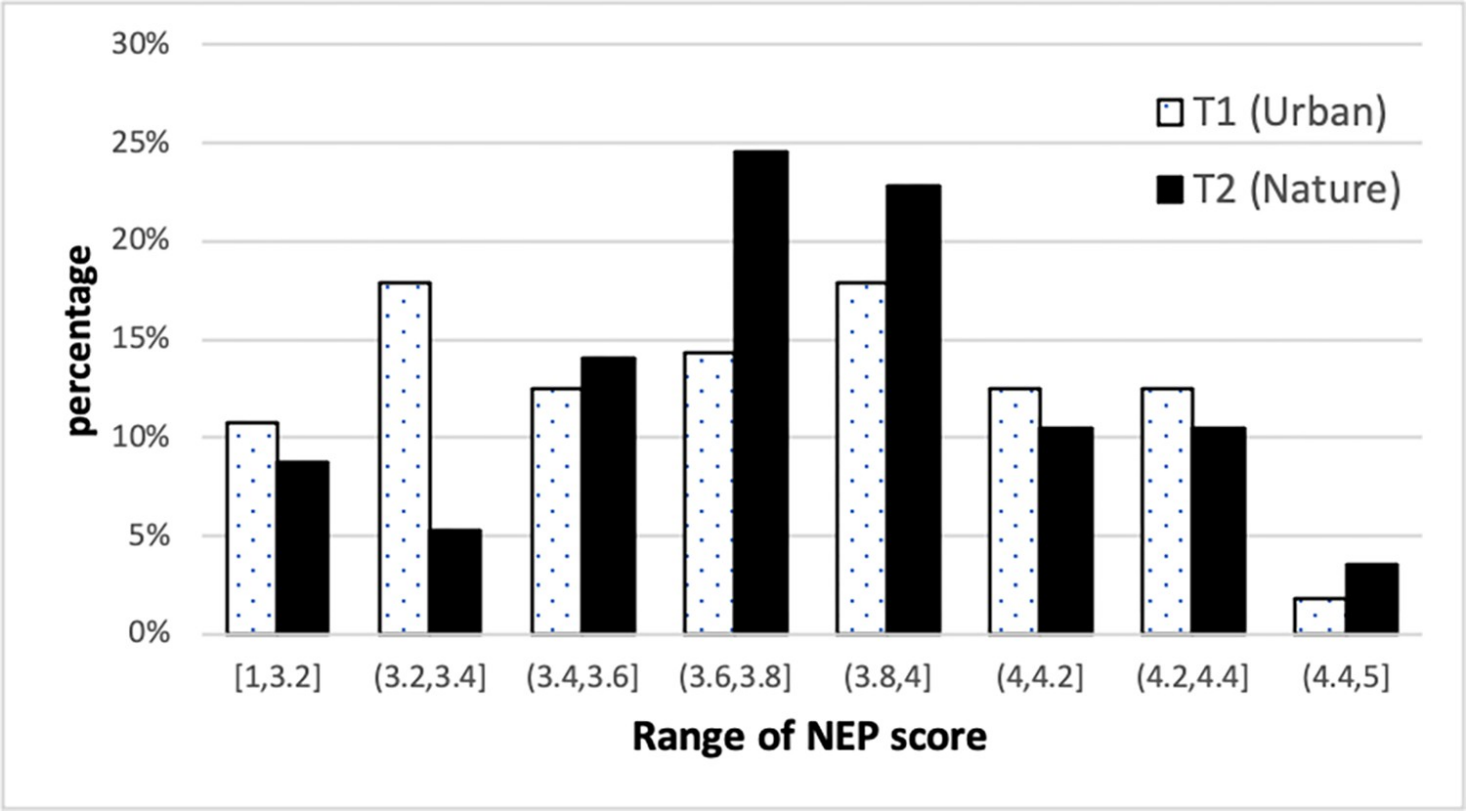
Range of average NEP score for Treatment T1 (Urban) and Treatment T2 (Nature).

We first gather the preliminary results on participants stated environmental beliefs through the NEP scale. Even if average NEP scores are similar for both the urban and nature treatments, the distributions are not similar; we observe a flatter distribution with more extreme outliers in the urban treatment and a more asymmetric distribution in the nature treatment (see Fig 1). In order to control for the skewness and kurtosis of distributions, in the subsequent analysis we created a dummy variable for the NEP score using the Johnson–Neyman procedure [54]. We distinguish participants with a low level of environmental beliefs (*NEP-Low*), i.e., a score lower than 4, from those with a high level (*NEP-High*), a score equal to or greater than 4 [13]. We may observe a higher share of subjects with high environmental values in the urban treatment– 37.5% of participants have an average score equal to or greater than 4 in the urban treatment, whereas roughly 30% have an average score equal to or greater than 4 in the nature treatment–(Tables 1 and 2).

**Table 1:** Share of *NEP-Low* (< 4) and *NEP-High* (≥ 4) subjects per treatment (in %).

	*NEP-Low* (NEP score < 4)	*NEP-High* (NEP score ≥ 4)
**Urban (T1)**	62.5%	37.5%
**Nature (T2)**	70.2%	29.8%

**Table 2:** NEP score, *NEP-Low* score and *NEP-High* score description (by treatment).

	NEP score on average	*NEP-Low* score on average	*NEP-High* score on average
**Urban (T1)**	3.75 (0.44)	3.48 (0.35)	4.19 (0.18)
**Nature (T2)**	3.81 (0.39)	3.62 (0.28)	4.25 (0.19)
**Wilcoxon-Mann-Whitney equality of population test** **(*p*-value)**	0.495	0.788	0.390

Wilcoxon-Mann-Whitney equality-of-populations rank test; standard deviations (*SD*) in parentheses; significant levels

*** *p*<0.01

** *p*<0.05

* *p*<0.1.

We then check for the impact of the videos on pleasure and emotional arousal. We use multiple hypothesis testing [55] and find a significant change in subjects’ pleasure and arousal after watching the video. Participants in the nature treatment experience a joint positive pleasure and arousal, suggesting that they feel a greater positive emotional state unlike the relative calmness commonly identified in studies that have found that nature induces a neutral emotional state (e.g., [56]). Participants in the urban treatment feel significantly more unpleasant (negative valence) with less emotional intensity (negative arousal) than participants in the nature treatment, with a difference in mean change at 18.69*** (*SD =* 3.81) and 8.34** (*SD =* 5.08), respectively.

**Fig 2:**
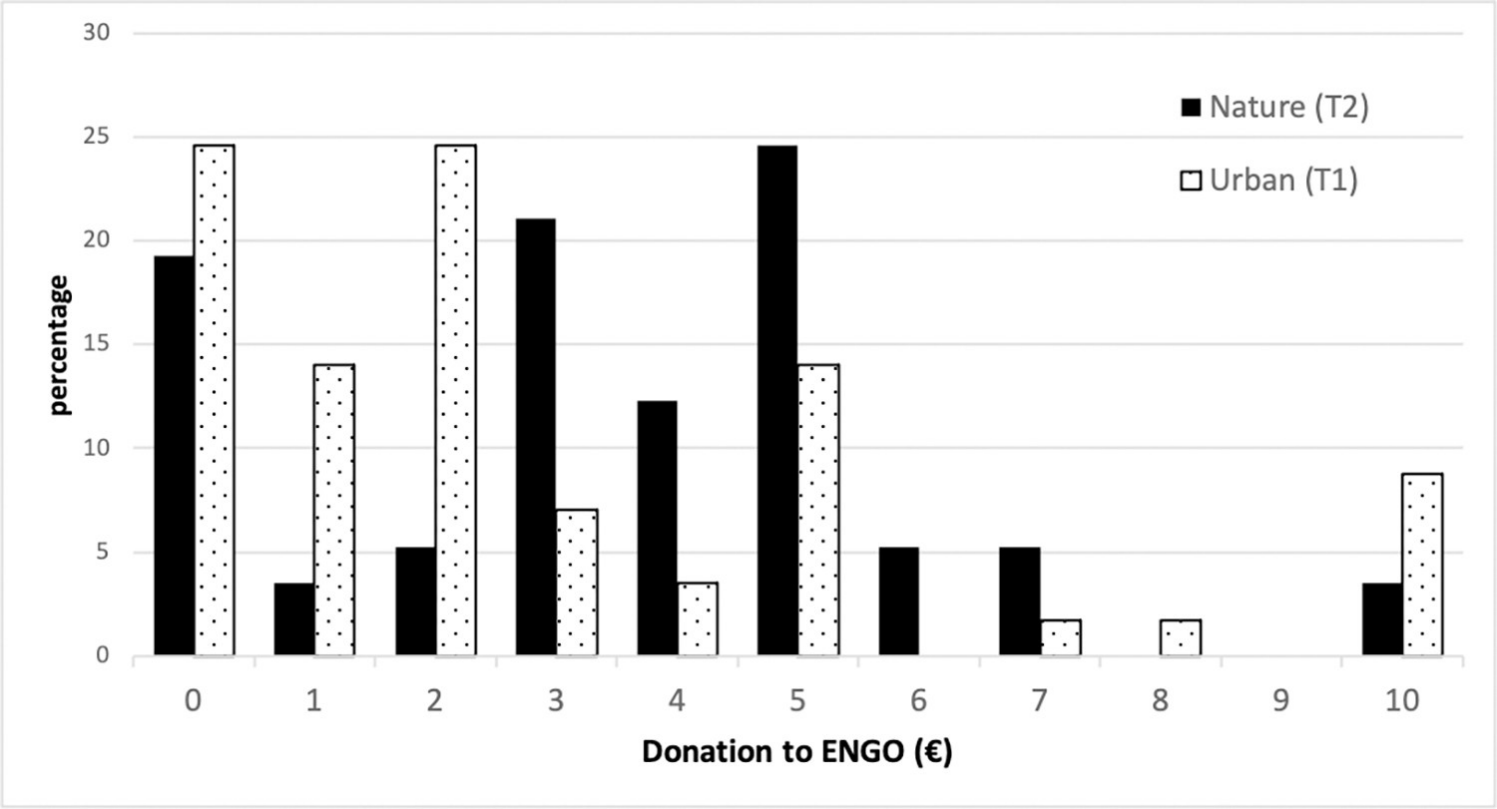
Range of donation to ENGO (€), by treatment (Urban (T1) and Nature (T2)).

Regarding eco-donations and eco-actions, we use descriptive statistics to compare the between-subject treatments using Wilcoxon–Mann–Whitney’s equality-of-populations rank test. First, for each treatment, we compare the average amounts donated, percentage of subjects who do not donate to an ENGO, and percentage of recycled headphone coverings (Table 3). To supplement this information on the average amounts donated, Fig 2 displays the range of donations in our choice set by treatment (€0–10).

**Table 3:** Eco-donation and eco-action decision description (by treatment).

	Number of observations	Amount given on average (€)	Share of subjects who do not donate anything (%)	Recycled hygienic headphone coverings (%)
**Urban (T1)**	56	€2.70 (€2.82)	25%	46.40%
**Nature (T2)**	57	€3.53 (€2.43)	19.30%	61.40%
**Wilcoxon-Mann-Whitney equality of population test** **(*p*-value)**	-	0.02**	-	0.11

Wilcoxon-Mann-Whitney equality-of-populations rank test; standard deviations (*SD*) in parentheses; significant levels: *** *p*<0.01, ** *p*<0.05, * *p*<0.1.

According to the average amounts donated and distributions, subjects exposed to the natural setting donate a significantly higher amount on average (T2: €3.53) than those exposed to the urban setting (T1: €2.70). Subjects who decide to keep the entire endowment for themselves (i.e., €0 donation) range from 19.3% (T2) to 25% (T1), which is lower than the figures found in the literature on dictator games (e.g., 36.11% in Engel’s [50] meta-analysis). Moreover, we observe that the distribution of donations differs for the two treatments (Fig 2), with a far higher frequency of high contributions for subjects who viewed the nature video (T2). Regarding the recycling of headphone coverings, the highest percentage (61.4%) is recycled by those exposed to the nature setting (T2) compared with the urban setting (T1) (46.4%).

To finetune our analysis, Table 4 provides a similar reading grid to distinguish *NEP-Low* participants from *NEP-High* participants. As stated in Section 3.2, the NEP scale allows us to measure an individual’s degree of endorsement to their ecological worldview by soliciting information indicative of the status they give to nature and the surrounding environment. *NEP-Low* individuals donate a significantly higher amount on average under the exposure to nature treatment (T2: €3.78) than under the urban exposure treatment (T1: €2.31). By contrast, *NEP-High* individuals contribute less after exposure to the natural setting (T2: €2.94) than after exposure to the urban setting (T1: €3.33), although this is not statistically significant. For the share of *NEP-Low* subjects behaving as purely egoistic dictators (i.e., €0 donation), this percentage is lower in T2 (15%) than in T1 (22.9%), whereas these percentages are reversed and close for *NEP-High* subjects in both settings (T1: 28.57%; T2: 29.41%). Finally, the percentage of recycled headphone coverings is more marked for *NEP-Low* individuals: 55% after the exposure to nature treatment (T2) compared with 28.57% by those exposed to the urban setting treatment (T1). The recycling rates are high for *NEP-High* individuals independently of the video watched (T1: 76.19%; T2: 76.47%).

**Table 4:** Differences in behavior between *NEP-Low* and *NEP-High* subjects (by treatment).

	*NEP-Low* (NEP score < 4)				*NEP-High* (NEP score ≥ 4)			
	Number of observations	Amount given on average (€)	Share of subjects who do not donate anything (%)	Recycled hygienic headphone coverings (%)	Number of observations	Amount given on average (€)	Share of subjects who do not donate anything (%)	Recycled hygienic headphone coverings (%)
**Urban (T1)**	35	€2.31 (€2.39)	22.90%	28.57%	21	€3.33 (€3.40)	28.57%	76.19%
**Nature (T2)**	40	€3.78 (€2.27)	15%	55%	17	€2.94 (€2.75)	29.41%	76.47%
**Wilcoxon-Mann-Whitney equality of population test** **(*p*-value)**	-	0.002***	-	0.022**		0.799	-	0.980

Wilcoxon-Mann-Whitney equality-of-populations rank test; standard deviations (*SD*) in parentheses; significant levels

*** *p*<0.01

** *p*<0.05

* *p*<0.1.

Overall, the descriptive data analysis shows that virtual exposure to nature increases average donations to the ENGO, reduces the probability of being purely egoistic, and increases recycling behavior, which supports Hypothesis 1: being exposed to a virtual natural setting increases PEB. Nevertheless, the environmental beliefs of participants are an important determinant of the way PEB evolves. Indeed, the increase in PEB (i.e., both donations to ENGOs and the recycling rate) after having been exposed to nature only occurs for participants with low environmental beliefs. Participants with high environmental beliefs behave similarly in both treatments, which does not lend support to Hypothesis 3. To complete this descriptive part, we now turn to the econometric analysis to refine our results.

### Econometric analysis

#### Eco-donations

With regards to eco-donations, to analyze more in-depth the role played by exposure to nature leading to the monetary donations to ENGOs, we control for individual heterogeneity by adding in socio-demographic control variables (e.g., age, gender, education), in using a set of econometric models to broadly assess the participation dimension (being a donor) as well as the amount donated (effective donation). We use Tobit estimates, as the Tobit regression model is a censored regression linked to our choice space and distribution (Table 5). We then refine our analysis by separating the mechanisms of the intensive margin of donating (i.e., likelihood of donating) from those of the extensive margin (i.e., level of donation). For this purpose, we use a Cragg–Hurdle regression model in which the lower bound 0 is considered as observed [33,50,57,58]. This is thus a two-stage decision with the combination of a Probit model explaining the factors driving the donation decision (Hurdle 0/1; Table 5) and a linear regression on donations, conditional on being a donor (Hurdle +; Table 5). In other words, we disentangle the participation and quantity dimensions in the monetary donation process within this procedure. This type of model is preferred to a two-stage selection model in which the analysis focuses on positive donations in the second stage [59]. Lastly, we compute the marginal effects to address the effective monetary impacts as the conditional mean estimates from the significant explanatory variables used in both stages of the Cragg–Hurdle model [60] (Table 5). The eco-donation econometric analysis without the sociodemographic controls is provided in Table S3.1 in S1 Table. All the econometric analyses were conducted using the STATA software (16.0). The STATA commands were respectively *tobit* to run the Tobit regression model *churdle* to run the Cragg–Hurdle regression model, *margins* to derive the marginal effects in terms of the conditional mean estimates from the significant explanatory variables, and *outreg2* to provide the results. The results are provided at the 1% (***), 5% (**), and 10% (*) significance levels.

**Table 5:** Treatment effects, intensive and extensive margins of monetary donation, and effective monetary impacts (€).

VARIABLES	Tobit	Cragg-Hurdle		
		Hurdle 0/1 *Likelihood*	Hurdle + *Regression*	Effective monetary impacts (€) *Marginal effects*
***Nature (T2)***	1.652**	0.271	2.138**	1.481**
	(0.743)	(0.363)	(0.881)	(0.605)
***Urban (T1)***	*Ref*.	*Ref*.	*Ref*.	
***Gender (Male)***	-1.076*	-0.859***	0.116	-0.761*
	(0.613)	(0.330)	(0.665)	(0.478)
***Age***	0.088	0.050	0.050	0.0790
	(0.079)	(0.047)	(0.079)	(0.064)
***Student***	-1.495	-0.797	-0.576	-1.134
	(1.029)	(0.588)	(1.060)	(0.828)
***NEP-High***	0.626	-0.333	2.144**	0.865
	(0.892)	(0.406)	(1.030)	(0.795)
Nature (T2) * NEP-High	-2.146*	-0.607	-2.494*	
	(1.309)	(0.608)	(1.467)	
***Constant***	1.925	1.023	1.317	
	(2.362)	(1.320)	(2.439)	
***lnsigma*, *Constant***	-	0.955***(0.113)		
***/sigma***	3.060 (0.243)	2.600 (0.294)		
**LL**	-247.454	-241.370		
**LR Chi** ^**2**^ **(6)**	13.13**	23.43***		
**Pseudo R** ^**2**^	0.026	0.046		
**Number of observations**	113	113		
**Session controls**	Yes	Yes		

Standard errors in parentheses; significant levels

*** *p*<0.01

** *p*<0.05

* *p*<0.1.

Considering the urban surrounding (T1) as the reference, the censored regression estimates show that exposure to a virtual natural setting has a more positive impact on donations to ENGOs (*Nature (T2)*, 1.652**) than exposure to the virtual urban setting. However, this positive impact is canceled out for individuals with high environmental beliefs (*Nature (T2)* times *NEP-High*, -2.146*). In other words, exposure to a virtual natural setting has no significant impact on participants with high environmental beliefs. If we break down the intensive margin of donating from the extensive margin, the Cragg–Hurdle model shows that the impacts of exposure to nature and environmental beliefs are positive on donation levels at the extensive margin (*Nature (T2)*, 2.138**; *NEP-High*, 2.144**); more precisely, the impact of exposure to nature is lessened for individuals with high environmental beliefs (*Nature (T2)* times *NEP-High*, -2.494*). We also observe a gender effect: men donate less than women (*Gender (Male)*, -1.076*). The main explanation of this decrease is the likelihood of donating, which is lower for men than for women (*Gender (Male)*, -0.769***). One explanation of this result might be the fact that environmental protection and behavior are considered feminine activities in which men are reluctant to engage to maintain their gender identity [61]. Nevertheless, considering solely men in our sample, they give significantly more in the nature treatment and are then sensitive to nature exposure (see Table S3.2 in S1 Table). Estimating the marginal effects from the Cragg–Hurdle model in terms of the effective monetary impact allows us to conclude that exposure to nature results in a €1.48 greater donation than in the urban exposure treatment on average (*Nature (T2)*, 1.481**). We find no statistically significant impact for gender and having high environmental beliefs.

Consequently, our econometric analysis of eco-donations confirms Hypothesis 1 as virtual exposure to a natural setting leads individuals to act in a pro-environmental manner by increasing eco-donations and rejects Hypothesis 3 as virtual exposure to a natural setting positively affects only individuals with a low level of environmental beliefs.

#### Eco-actions

With regards to eco-actions, we analyze the mechanisms behind the recycling decision (Table 6). We use Probit estimates, as the Probit regression model is a binomial regression that assesses the factors behind the probability of recycling that allows us to identify the marginal effects in terms of percentage points to check for spillover effects from the eco-donation decision to the eco-action one. The eco-action econometric analysis without the sociodemographic controls is provided in Table S3.3 in S1 Table. The STATA commands used were *probit* to run the binomial regression model, *margins* to derive the marginal effects in terms of probability points from the significant explanatory variables, and *outreg2* to provide the results.

**Table 6:** Binomial regression estimates and marginal effects.

VARIABLES	Probit 0/1 *Likelihood*	Marginal effects *Probability points*
***Nature (T2)***	0.841***	0.279**
	(0.318)	(0.095)
***Urban (T1)***	*Ref*.	
***Donation (Yes)***	-0.041	-0.013
	(0.323)	(0.107)
***Gender (Male)***	0.064	0.021
	(0.271)	(0.090)
***Age***	-0.083**	-0.028**
	(0.036)	(0.011)
***Student***	-0.199	-0.066
	(0.485)	(0.161)
***NEP-High***	1.500***	0.478***
	(0.402)	(0.091)
Nature (T2) * NEP-High	-1.016*	
	(0.576)	
***Constant***	1.389	
	(1.104)	
**LL**	-66.157	
**LR Chi** ^**2**^ **(7)**	23.62***	
**Pseudo R** ^**2**^	0.152	
**Number of observations**	113	
**Session controls**	Yes	

Standard errors in parentheses; significant levels

*** p<0.01

** p<0.05

* p<0.1.

First, there is no direct spillover effect. We observe that donating to an ENGO does not have a significant impact on recycling behavior (*Donation (Yes)*, -0.041). Although this is not significant, the sign is negative and suggests a self-licensing phenomenon [37]. By contrast, participants exposed to the natural setting (*Nature (T2)*, 0.841***) are more likely to recycle than those exposed to the urban setting (T1). This is reflected in the marginal effects in terms of percentage points: the probability of performing a green deed rises under exposure to nature (*Nature (T2)*, 0.279***) compared with the urban exposure treatment. Further, participants with high environmental beliefs are more likely to recycle than those with low levels, regarding both the likelihood of recycling (*NEP-High*, 1.500**) and the probability of performing it in terms of percentage points (*NEP-High*, 0.448***). Interestingly, the likelihood of participants with high environmental beliefs recycling is mitigated for those exposed to the natural setting (Nature (T2)*NEP-High, -1.016*). Moreover, older people are less likely to recycle and then perform the green deed.

Our econometric analysis of eco-actions reconfirms Hypothesis 1: being exposed to a natural surrounding leads individual to act in a pro-environmental manner by increasing the likelihood of them performing an eco-action. However, we are unable to support Hypotheses 2 and 3. First, we do not observe any spillover effect between PEB under virtual exposure to nature; that is, donating to the ENGO does not increase or decrease the likelihood of recycling after having been exposed to a virtual natural environment. Second, exposure to nature decreases the likelihood that participants with high environmental beliefs will recycle.

## Robustness checks

### The emotional impact of nature video exposure

To ensure that the impact of the virtual natural setting is not linked to any conveyed positive emotional state, we introduced a supplementary control treatment (T3) inducing positive emotions not linked to nature [62,63]. In Treatment T3 (“positive emotions”), participants view a succession of scenes from four films selected from Uhrig et al. [64]; the four films are *Bruce Almighty*, *Sister Act*, *What Women Want*, and *Wall-E*.

The experimental conditions and procedure are the same as for T1 and T2 (e.g., between-subjects design that excludes a comparison of the video content across conditions, 12-minute duration, pleasure, and arousal measurement). This additional treatment took place in the LEEM by running three additional sessions between December 10 and December 12, 2018 and applying the same procedures as for the previous treatments (ORSEE, Python programming for running the sessions, single-blind experiment, consent was informed and subjects provided written consent to participate, experimental steps, duration, and average earnings). 60 subjects were randomly recruited for this treatment.

Comparing the nature treatment (T2) with the positive emotions treatment (T3), subjects are similar in age (T2: *M*_*Age*_ = 22.74 years, *SD =* 4.13; T3: *M*_*Age*_ = 22.33 years, *SD* = 4.81). There is an overrepresentation of men in T3 (63.33%) compared with T2 (49.12%), but the difference is not statistically significant (*X*^2^ = 1.756; *p* = 0.185). Both treatments are statistically similar in terms of the NEP scale score (T2: *M*_*NEP value*_ *=* 3.81, *SD =* 0.39; T3: *M*_*NEP value*_ *=* 3.69, *SD =* 0.49), with a comparable share of *NEP-Low* and *NEP-High* subjects (considering *NEP-High* subjects, T2: 29.82% and T3: 25%). Most subjects are full-time students for both treatments (T2: 93%; T3: 90%).

Considering the impact of the videos on pleasure and emotional arousal, there is no significant change in subjects’ pleasure or arousal after watching the video between the treatments (mean change at 1.78 (*SD =* 3.22) for pleasure and 0.99 (*SD =* 4.99) for arousal). Hence, importantly, participants in both treatments experience a similar level of positive pleasure and arousal after virtual exposure. Thus, T3 is a suitable control treatment for testing the impact of a positive emotional state on PEB.

**Fig 3:**
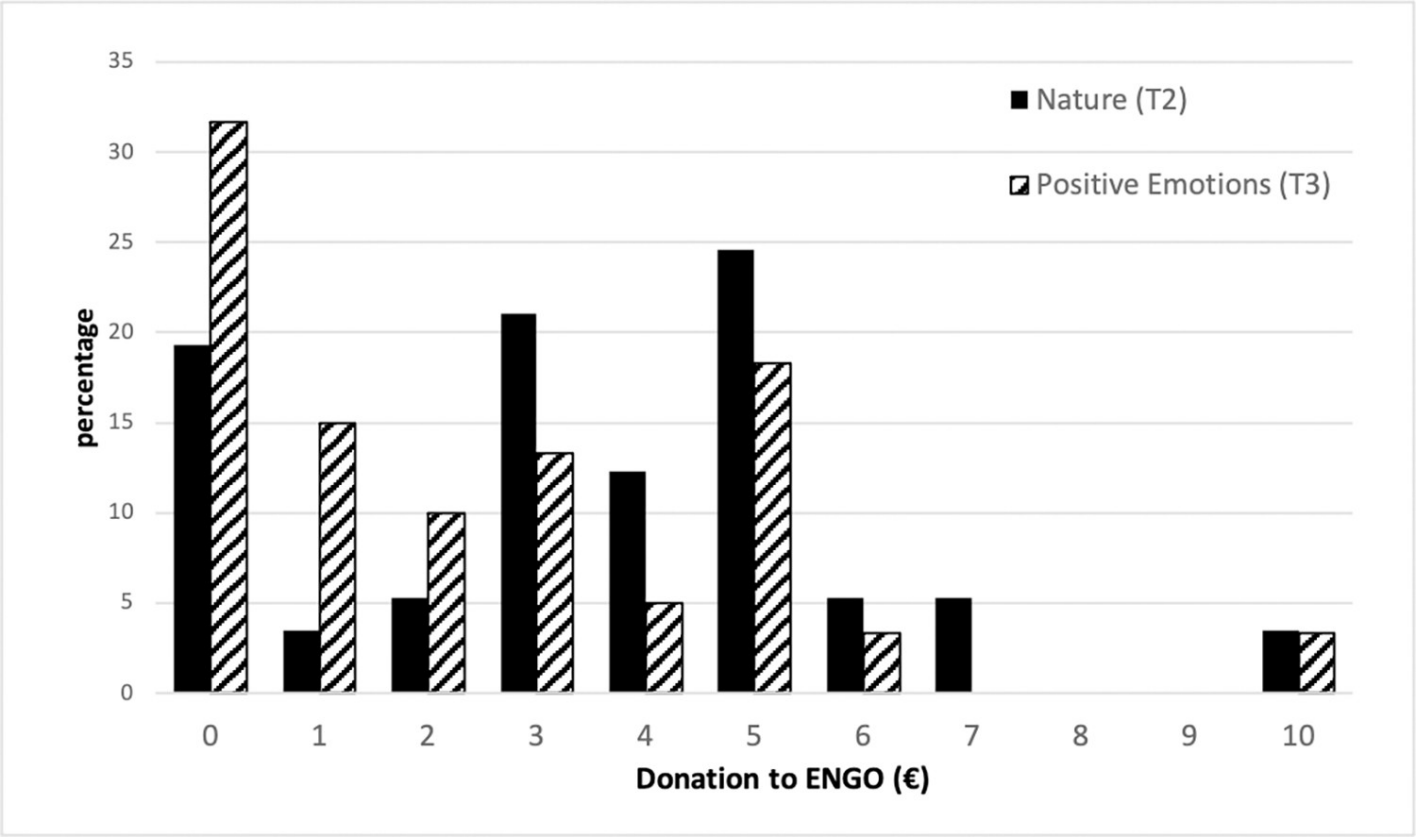
Range of donation to ENGO (€), by treatment (Nature (T2) and Positive emotions (T3)).

According to the distributions and average amounts donated (Table 7 and Fig 3), subjects exposed to the natural setting donate a significantly higher amount on average (T2: €3.53) than those exposed to the positive emotions setting (T3: €2.40) (Wilcoxon–Mann–Whitney’s equality-of-populations rank test). The proportion of participants who do not donate to the ENGO is greater in T3 (31.66%) than in T2 (19.3%). With regards to the recycling of headphone coverings, there is no significant difference between the treatments (T2: 61.4%; T3: 50%).

**Table 7:** Eco-donation and eco-action decision description (Treatments T2 and T3).

	Number of observations	Amount given on average (€)	Share of subjects who do not donate anything (%)	Recycled hygienic headphone coverings (%)
**Nature (T2)**	57	€3.53 (€2.43)	19.30%	61.40%
**Positive emotions** **(T3)**	60	€2.40 (€2.45)	31.66%	50%
**Wilcoxon-Mann-Whitney equality of population test** **(*p*-value)**		0.01***	-	0.217

Wilcoxon-Mann-Whitney equality-of-populations rank test; standard deviations (*SD*) in parentheses; significant levels

*** *p*<0.01

** *p*<0.05

* *p*<0.1.

The econometric analysis shows that virtual exposure to a natural setting leads individuals to act in a pro-environmental manner by increasing eco-donations regardless of the positive emotional content of the exposure (Table 8). Indeed, the Tobit estimates show that virtual exposure to a natural surrounding has a strong positive impact on donation levels to ENGOs (*Nature (T2)*, 1.818***) compared with exposure to solely positive emotions. This depicts that the impact of exposure to the nature video is not linked to its emotional content. We also note a gender effect, with men contributing less independently of the exposure (*Gender (Male)*, -1.137*). Concerning eco-actions, the Probit estimates show that virtual exposure to a natural surrounding does not have a significant impact on the likelihood of recycling (*Nature (T2)*, 0.081) compared with the “positive emotions” exposure treatment.

**Table 8:** Robustness check: Emotional impact (Treatments T2 and T3).

VARIABLES	Eco-donation	Eco-action
	
	Tobit	Probit 0/1 *Likelihood*
***Nature (T2)***	1.818***	0.081
	(0.688)	(0.282)
***Positive emotions (T3)***	*Ref*.	*Ref*.
***Donation (Yes)***	-	-0.101
		(0.283)
***Gender (Male)***	-1.137*	-0.164
	(0.607)	(0.254)
***Age***	0.038	0.019
	(0.660)	(0.028)
***Student***	-0.207	0.020
	(1.117)	(0.452)
***NEP-High***	0.622	-0.134
	(0.958)	(0.384)
Nature (T2) * NEP-High	-2.018	0.700
	(1.335)	(0.558)
***Constant***	1.699	-0.231
	(2.135)	(0.868)
***/sigma***	3.013 (1.212)	-
**LL**	-248.141	-77.857
**LR Chi** ^**2**^ **(6)**	12.04*	-
**LR Chi** ^**2**^ **(7)**	-	5.03
**Pseudo R** ^**2**^	0.024	0.031
**Number of observations**	117	117
**Session controls**	Yes	Yes

Standard errors in parentheses; significant levels

*** p<0.01

** p<0.05

* p<0.1.

### The recipient status

To ensure that the impact of the virtual natural setting is not driven by any demand effect, we control for this dimension by introducing a fourth treatment (T4) in which the recipient is a humanitarian charity as opposed to an environmental one (T1, T2, and T3). In T4, subjects view the same documentary on Yellowstone Park as in T2 and are invited to play a modified dictator game in which the recipient is a humanitarian non-governmental organization (HNGO) instead of an ENGO.

Each participant is given a €10 endowment and indicates the amount (an integer between €0 and €10) to donate to an HNGO. To avoid any anchoring effect and cover international, national, and local actions, participants could choose among four HNGOs (similar to the four ENGOs in T1, T2, and T3): *The French Red Cross* (one of the leading humanitarian movements worldwide), *Le Secours Populaire français* (a French non-political organization), *Action contre la Faim* (a French non-political organization), and *Crocos du monde* (a student union promoting humanitarian causes created in 2007 at the University of Montpellier–Nîmes).

Once again, the experimental conditions are replicated and the same as for the other experimental treatments (e.g., between-subjects design that excludes video content comparison across conditions, 12-minute duration, pleasure, and arousal measurement). This additional treatment took place in the LEEM in sessions run on September 10 and September 18, 2020, applying the same procedures as for previous treatments (ORSEE, Python programming for running the sessions, single-blind experiment, consent was informed and subjects provided written consent to participate, experimental steps (except for the NGO choice; see instructions in S1 File), duration, and average earnings). Altogether, 49 subjects were randomly recruited for this treatment.

Comparing the nature treatment (T2) with the nature HNGO treatment (T4), subjects are similar in age (T2: *M*_*Age*_ = 22.74 years, *SD =* 4.13; T4: *M*_*Age*_ = 21.04 years, *SD* = 4.84). There is an underrepresentation of men in the nature HNGO treatment (T4) (40.82%) compared with the nature treatment (T2) (49.12%) but the difference is not statistically significant (*X*^2^. = 0.540; *p* = 0.462). Most subjects are full-time students (T2: 93%; T4: 75.51%). Both treatments are statistically similar in terms of the NEP scale score (T2: *M*_*NEP value*_ *=* 3.81, *SD =* 0.39; T4: *M*_*NEP value*_ *=* 3.82, *SD =* 0.46), with a higher share of *NEP-High* subjects in T4 (T2: 29.82%; T4: 53.06%). As T4 took place in September 2020, we can assume that the Covid-19 pandemic affected salience toward environmental issues.

**Fig 4:**
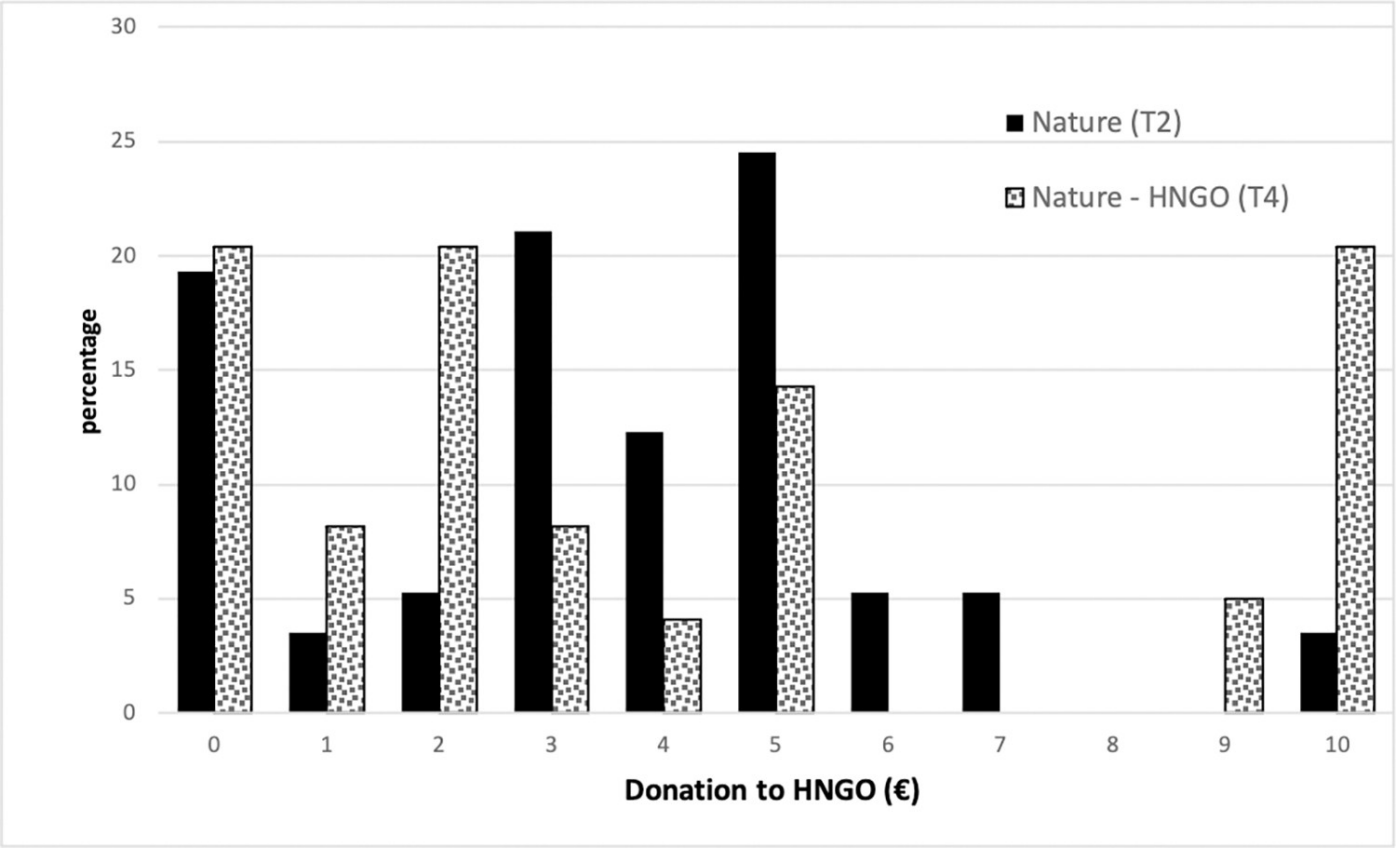
Range of donation to ENGO (€), by treatment (Nature (T2) and Nature HNGO (T4)).

According to the distributions and average amounts donated (Table 9 and Fig 4), subjects exposed to the natural setting in T2 donate a lower amount on average (€3.53) than those exposed to the nature setting in T4 (€4.02), although this is not statistically significant (Wilcoxon–Mann–Whitney’s equality-of-populations rank test). The proportions of participants who donate nothing are similar in both treatments (T2: 19.3%; T4: 20.41%). There is a higher share of very high contributors (€9 and €10) in T4 (Fig 4). With regards to the recycling of headphone coverings, the share is greater in T4, although there is no significant difference between the treatments (T2: 61.4%; T4: 73.5%). As T4 was run during the Covid-19 pandemic, it is unsurprising that participants were adopting more hygiene-related behaviors [65].

**Table 9:** Eco-donation and eco-action decision description (Treatments T2 and T4).

	Number of observations	Amount given on average (€)	Share of subjects who do not donate anything (%)	Recycled hygienic headphone coverings (%)
**Nature (T2)**	57	€3.53 (€2.43)	19.30%	61.40%
**Nature HNGO (T4)**	49	€4.02 (€3.70)	20.41%	73.50%
**Wilcoxon-Mann-Whitney equality of population test** **(*p*-value)**		0.887	-	0.190

Wilcoxon-Mann-Whitney equality-of-populations rank test; standard deviations (*SD*) in parentheses; significant levels: *** *p*<0.01, ** *p*<0.05, * *p*<0.1.

The econometric analyses show no differences in eco-donations and eco-actions between T2 and T4 (Table 10). In other words, virtual exposure to a natural setting impacts prosocial behavior and PEB in a similar way both in terms of monetary decisions and in terms of recycling. Hence, the impact of a virtual natural setting on donations to ENGOs is not driven by any demand effect from our experimental protocol.

**Table 10:** Robustness check: Recipient status (Treatments T2 and T4).

VARIABLES	Eco-donation	Eco-action
	Tobit	Probit 0/1 *Likelihood*
***Nature (T2)***	-0.327	-0.367
	(0.991)	(0.354)
***Nature HNGO (T4)***	*Ref*.	*Ref*.
***Donation (Yes)***	-	-0.097
		(0.336)
***Gender (Male)***	-0.348	-0.179
	(0.739)	(0.267)
***Age***	0.060	-0.028
	(0.086)	(0.032)
***Student***	1.085	-0.481
	(1.124)	(0.430)
***NEP-High***	0.297	-0.120
	(1.071)	(0.392)
Nature (T2) * NEP-High	-1.344	0.546
	(1.559)	(0.562)
***Constant***	1.565	1.785*
	(2.376)	(0.923)
***/sigma***	3.625 (1.456)	-
**LL**	-251.782	-64.114
**LR Chi** ^**2**^ **(6)**	3.11	-
**LR Chi** ^**2**^ **(7)**	-	6.25
**Pseudo R** ^**2**^	0.006	0.047
**Number of observations**	106	106
**Session controls**	Yes	Yes

Standard errors in parentheses; significant levels: *** p<0.01, ** p<0.05, * p<0.1.

### Sensitivity of donations to NEP values under virtual exposure to nature

**Fig 5:**
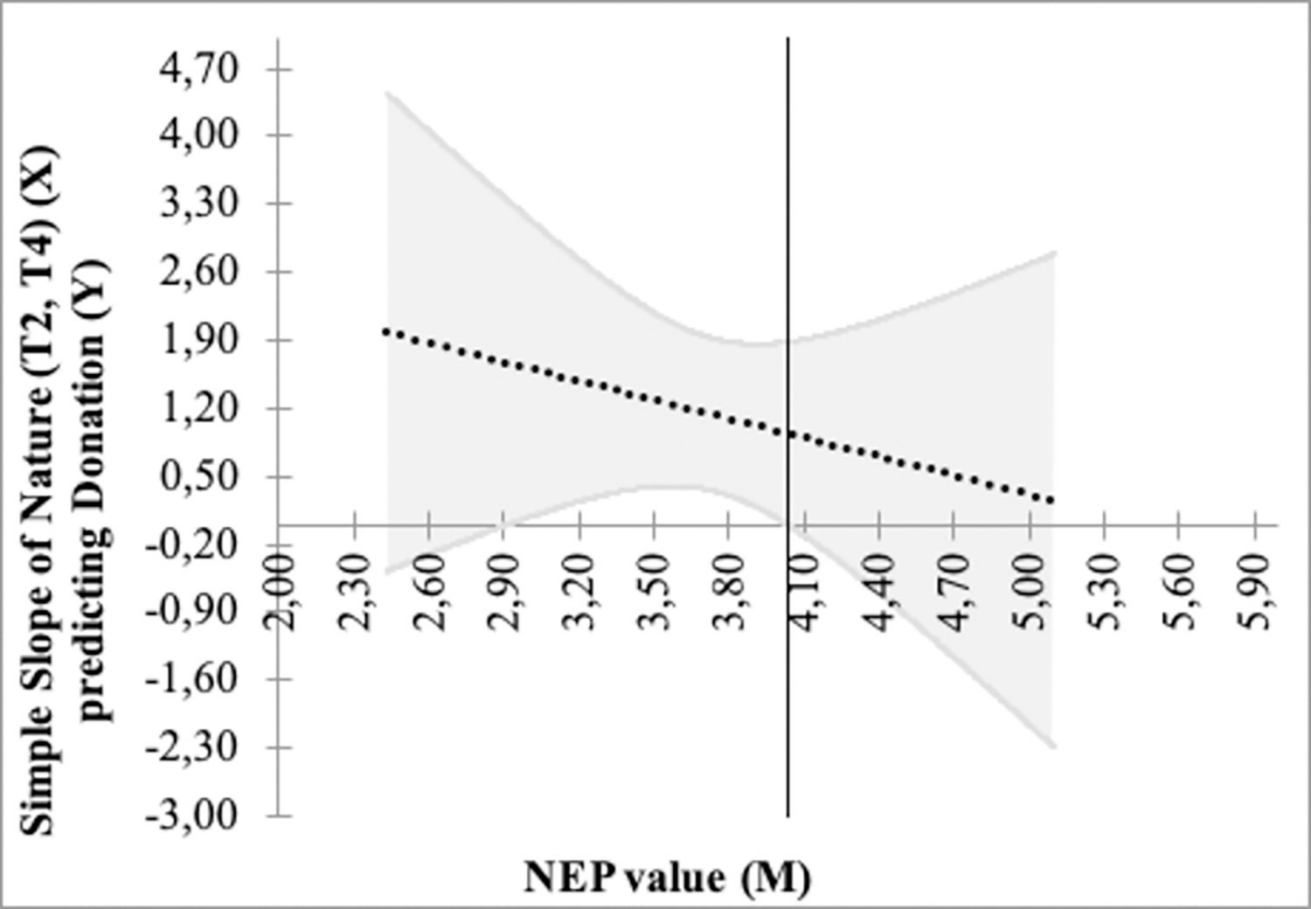
Relation between donation and virtual exposure to nature and the NEP value.

To analyze the sensitivity of our results linked to our cut-off set at 4 to separate *NEP-Low* from *NEP-High* participants, we apply the Johnson–Neyman procedure that allows for a moderation analysis [54]. The moderator is then the mean NEP value, whereas donation is explained by the treatment effect through virtual exposure to nature (focal predictor). We analyze the impact on donation (a continuous variable) unlike our green deed (a binary one). To ascertain a broader view of exposure to the nature video, we compare donations in the grouping observations from Treatments T2 and T4 (*n* = 106) with donations under the other types of video exposure in Treatments T1 and T3 (*n* = 116). The environmental and humanitarian causes refer to prosocial behaviors and the donations are not statistically different (see Section 5.2). Fig 5 shows the Johnson–Neyman graph for the model relating donation to virtual exposure to nature, the NEP value, and their interaction.

Fig 5 shows that the endogenous cut-off is at a mean NEP of 4.03. If the mean NEP value increases, the predicted relative donation level decreases under virtual exposure to nature, emphasizing the need to target individuals with low environmental beliefs to induce changes. Hence, our results are robust to the cut-off set in our experimental procedure.

## Discussion and conclusion

To supplement traditional methods employed by environmental policies, “greening” individual behaviors through non-monetary instruments and behavioral insights could be an essential component to mitigate climate change as well as help to solve the environmental problems caused by humans [66]. Behavioral-based interventions including green nudges [9] could guide individuals’ decisions and encourage them to adopt or reinforce PEB [67]. In this study, we analyze the extent to which a simple exposure to a virtual natural setting through video footage influences individuals’ PEB. Specifically, we examine two types of real decisions: a monetary decision through an eco-donation and a non-monetary decision through an eco-action.

Our results indicate that virtual exposure to nature boosts eco-donations and eco-actions. Our results are consistent with those of previous [32,33] and are in line with research on the short-term consequences of exposure to nature, which suggests that nature can promote sustainability and cooperative behaviors in the context of commons dilemmas [19,22,21,31]. However, our findings differ from those of Klein and Hilbig [18], who show that increasing the salience of nature per se does not increase PEB in the virtual exposure to nature setting, except when salience focuses on destroyed nature and the need for environmental protection. Interestingly, we find that an important driver of PEB is the individual’s level of environmental beliefs. More precisely, in the virtual exposure to nature setting, only individuals with low environmental beliefs reinforce their direct and indirect PEBs.

Another interesting result is the lack of any direct link between eco-donations and eco-actions in terms of spillover effects. In other words, both behaviors, corresponding to an indirect and a direct PEB, respectively [2], are positively impacted by virtual exposure to nature, but this exposure does not create any PEB dynamics.

We should emphasize the limitations of our experimental framework. First, nature videos are conventionally used as a neutral stimulus and considered as a control condition in experimental protocols [56], whereas our exposure features a positive emotional content. To a larger extent, we cannot exclude that our results are probably sensitive to our video choice, even though the choice of the urban video was done to make it similar enough in its conception to the one of nature, i.e., relatively aesthetic, but antithetical to nature. Moreover, another conventional control treatment would have been a treatment with no virtual exposure to both nature and urban videos. However, as we endowed the participants with hygienic headphone coverings for the audio headset for video viewing that was a part of the experiment / eco-action, we could not introduce an experimental treatment without any stimuli. Second, the environmental beliefs elicitation and nature videos may have primed environmental conservation and thus individuals’ observed behaviors. Third, in our socioeconomic demographic questionnaire, we did not ask participants about their personal experiences of the environment (e.g., childhood and current residence, environmental training, past actions), which could have affected their responsiveness.

To address these issues, we carry out several robustness checks. With regards to the positive emotional content induced by our nature video, we control for the emotional component by considering an experimental treatment that induces a similar positive emotional state but disconnected with nature. This comparison allows us to confirm that the increase in PEB is explained by the nature content of the video and not the emotional feature. Zelenski et al. [31] discuss this neutral dimension of nature and state that “‘neutrality’ may be unwisely assumed in some research contexts”. Our virtual exposure to nature is also in line with the real world, as it has been shown that nature induces a positive mood and emotional state [14,19,21,68].

Our between-subjects design, especially the introduction of an experimental treatment in which the recipient is an HNGO, allows us to control for any potential priming impact. Indeed, we show that indirect donations increase similarly for both ENGOs and HNGOs after being exposed to the virtual natural setting. In addition, we could have assessed participants’ environmental beliefs at the end of the experiment, although such an assessment could have been impacted by the video exposure and eco-donations in turn. However, we could not control for personal experiences of the environment ex post, and this could be interesting in future experimental manipulations.

Our findings open the door to designing policies that aim to expose people to nature or natural environments. More precisely, according to our findings, virtual exposure to nature is most effective when targeted at individuals with a low level of environmental beliefs. From this, we would also expect that visual cues featuring exposure to natural landscapes, environmentally friendly ways of life, and green working conditions could be promising policy tools [69,70].

Integrating exposure to nature in a broader manner to improve PEB could be operationalized by green nudge designs [9], which are straightforward, low-cost, and non-binding actions. More research is needed to study external validity. The implementation of green nudges that rely on natural features raises issues about the persistence of behavioral insights and potential waning effects. A one-shot exposure to nature may not permanently change a person’s behavior and momentary exposure to nature may not lead to sustainable choices in the same way that more consistent exposure might; indeed, it could even generate negative spillover effects [35]. Researchers could study this potential basis for future decisions and hence strengthen the original cause for PEB, as this would allow measuring cost-effectiveness and behavioral change over time [67].

## Supporting information

S1 Table Econometric analyses without socio-demographic controls. (DOCX)

S1 File Laboratory experiment instructions. (DOCX)

S2 File Socio-demographic questions. (DOCX)
