# Supplementary material for: The impact of nature video exposure on pro-environmental behavior: An experimental investigation
Source: PLoS One. 2022 Nov 8;17(11):e0275806. doi: 10.1371/journal.pone.0275806 (PMC9642880; doi:10.1371/journal.pone.0275806)
Supplement: S1 Table — (DOCX) [file pone.0275806.s001.docx]

**Supplementary material S3. Econometric analyses without socio-demographic controls**

**Table S3.1. Treatment effects, intensive and extensive margins of monetary donation, and effective monetary impacts (€)**

| **VARIABLES** | **Tobit** | **Cragg-Hurdle** | | |
| --- | --- | --- | --- | --- |
|  |  | **Hurdle 0/1**  ***Likelihood*** | **Hurdle +**  ***Regression*** | **Effective monetary impacts (€)**  ***Marginal effects*** |
|  |  |  |  |  |
| ***Nature (T2)*** | 1.669** | 0.293 | 2.129** | 1.532** |
|  | (0.749) | (0.337) | (0.871) | (0.609) |
| ***Urban (T1)*** | *Ref.* | *Ref.* | *Ref.* |  |
|  |  |  |  |  |
| ***NEP-High*** | 0.972 | -0.178 | 2.410** | 1.178 |
|  | (0.900) | (0.373) | (1.040) | (0.806) |
| ***Nature (T2) * NEP-High*** | -2.103* | -0.317 | -2.765* |  |
|  | (1.303) | (0.548) | (1.450) |  |
| ***Constant*** | 1.868*** | 0.744*** | 1.988** |  |
|  | (0.553) | (0.235) | (0.777) |  |
| ***lnsigma, Constant*** | - | 0.964***  (0.113) | |  |
| ***/sigma*** | 3.161  (0.252) | 2.623  (0.299) | |  |
| **LL** | -251.488 | -247.881 | |  |
| **LR Chi^2^(3)** | 5.06 | 10.40** | |  |
| **Pseudo R^2^** | 0.01 | 0.021 | |  |
| **Number of observations** | 113 | 113 | |  |
| **Session controls** | Yes | Yes | |  |

Standard errors in parentheses; significant levels: *** *p*<0.01, ** *p*<0.05, * *p*<0.1

**Table S3.2. Treatment effects, intensive and extensive margins of monetary donation, for men only**

| **VARIABLES** | **Tobit** | **Cragg-Hurdle** | |
| --- | --- | --- | --- |
|  |  | **Hurdle 0/1**  ***Likelihood*** | **Hurdle +**  ***Regression*** |
|  |  |  |  |
| ***Nature (T2)*** | 1.83* | 0.192 | 2.965** |
|  | (1.076) | (0.627) | (0.237) |
| ***Urban (T1)*** | *Ref.* | *Ref.* | *Ref.* |
|  |  |  |  |
| ***Age*** | 0.041 | 0.051 | -0.087 |
|  | (0.118) | (0.047) | (0.109) |
| ***Student*** | 2.442* | -0.790 | -0.378 |
|  | (1.421) | (0.591) | (1.238) |
| ***NEP-High*** | 1.365 | -0.334 | 4.101*** |
|  | (1.252) | (0.407) | (1.417) |
| ***Nature (T2) * NEP-High*** | -0.867 | -0.580 | -3.008 |
|  | (2.187) | (0.632) | (2.065) |
| ***Constant*** | 2.264 | 1.036 | 3.506 |
|  | (3.271) | (1.324) | (3.012) |
| ***lnsigma, Constant*** | - | 0.92***  (0.153) | |
| ***/sigma*** | 3.404  (0.386) | 2.509  (0.384) | |
| **LL** | -136.025 | -129.204 | |
| **LR Chi^2^(6)** | 8.06 | 20.44** | |
| **Pseudo R^2^** | 0.029 | 0.073 | |
| **Number of observations** | 64 | 64 | |
| **Session controls** | Yes | Yes | |

Standard errors in parentheses; significant levels: *** *p*<0.01, ** *p*<0.05, * *p*<0.1

**Table S3.3. Binomial regression estimates and marginal effects**

| **VARIABLES** | **Probit 0/1**  ***Likelihood*** | **Marginal effects**  ***Probability points*** |
| --- | --- | --- |
|  |  |  |
| ***Nature (T2)*** | 0.706** | 0.246** |
|  | (0.302) | (0.097) |
| ***Urban (T1)*** | *Ref.* |  |
|  |  |  |
| ***Donation (Yes)*** | -0.150 | -0.052 |
|  | (0.303) | (0.106) |
| ***NEP-High*** | 1.274*** | 0.437*** |
|  | (0.376) | (0.101) |
| ***Nature (T2) * NEP-High*** | -0.704 |  |
|  | (0.542) |  |
| ***Constant*** | -0.452 |  |
|  | (0.322) |  |
| **LL** | -69.144 |  |
| **LR Chi^2^(4)** | 17.64*** |  |
| **Pseudo R^2^** | 0.113 |  |
| **Number of observations** | 113 |  |
| **Session controls** | Yes |  |

Standard errors in parentheses; significant levels: *** p<0.01, ** p<0.05, * p<0.1
