# Supplementary material for: The impact of nature video exposure on pro-environmental behavior: An experimental investigation
Source: PLoS One. 2022 Nov 8;17(11):e0275806. doi: 10.1371/journal.pone.0275806 (PMC9642880; doi:10.1371/journal.pone.0275806)
Supplement: S1 File — (DOCX) [file pone.0275806.s002.docx]

**Supplementary material S1. Laboratory experiment instructions**

**Good morning / Good afternoon,**

The experiment you are about to participate in is intended for the study of decision-making. We ask you to read the instructions carefully. Once each of you has read the instructions, an experimenter will read them aloud.

All your answers will be treated anonymously. You will indicate your choices using the computer you are sitting in front of.

The experiment is composed of 2 parts. For each part, messages on the screen will tell you when you can complete the set of associated tasks or if it is appropriate to wait. Waiting intervals are sometimes introduced so that all people participating in the experiment can progress at the same pace.

Your remuneration will be paid at the end of the experiment. Your remuneration will be the sum of your possible gain in Part 2 of the experiment and the show-up fee of €5 which corresponds to transport expenses. This sum will be given to you in cash at the end of the experiment, in Room C217 and on presentation of your identity card.

We ask you to remain totally silent during the entire experiment and not to give any signs that could influence your neighbors. Your cell phones must be turned off. You should not consult any documents other than those that have been distributed to you or that are presented to you on the computer screen.

If you have any questions, raise your hand and an experimenter will come and answer you in private.

I have read and accept the conditions of the experiment

***PART 1 INSTRUCTIONS***

The first part is composed of 3 tasks.

**Task 1:**

We ask you to rate your level of pleasure (from "not pleased" to "very pleased") and your state of arousal (from "very calm” to "very awake"). To do this, you have a cursor to move horizontally to express how you feel [47].


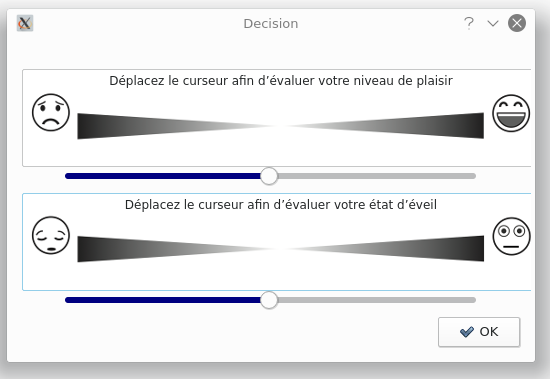


**Task 2:**

***For Treatments T1, T2 and T3***

You will be associated with an environmental non-governmental organization (ENGO) for the duration of the experiment. You can choose one of the following four ENGOs:

- *WWF* (*World Wildlife Fund*) (the world's leading nature conservation organization);
- *Fondation pour la Nature et l'Homme* (French, non-political organization);
- *France Nature Environnement* (French federation of organizations for the protection of nature and the environment);
- *Ouvre-Tête Alternative Sociale et Solidarité Écologique* (a student union created in 2006 at the University of Montpellier).

If needed, you will find at the end of these instructions a short presentation of these ENGOs.

***Alternatively, for Treatment T4***

You will be associated with a humanitarian non-governmental organization (HNGO) for the duration of the experiment. You can choose one of the following four HNGOs:

- *The French Red Cross* (one of the leading humanitarian movement worldwide);
- *Le Secours Populaire français* (a French, non-political organization);
- *Action contre la Faim* (a French, non-political organization);
- *Crocos du monde* (a student union promoting humanitarian causes created in 2007 at the University of Montpellier – Nîmes).

If needed, you will find at the end of these instructions a short presentation of these HNGOs.

**Task 3:**

We will present you 15 statements which concern the relationship between people and the environment. For each statement, we ask you to indicate whether you “Totally agree”, “Agree”, “Neither agree or disagree”, “Not agree” or “Not agree at all”. Please answer as sincerely as possible. We are interested in your opinion. There are no right or wrong answers.


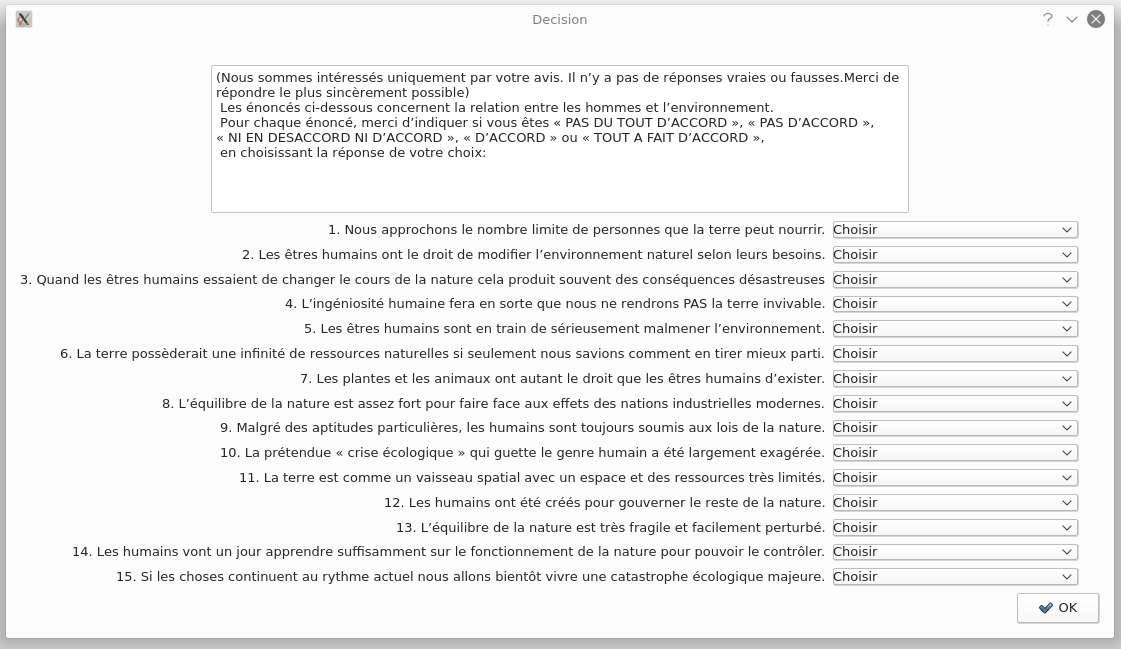


***PART 2 INSTRUCTIONS***

The second part is composed of 3 tasks.

**Task 1:**

A video will be presented to you. We ask you to look at it carefully. For this purpose, an audio headset is available with headphone protectors in the form of hygienic headphone coverings. We will ask you to throw them away at the end of the experiment; a dedicated bin will be available at the time of payment.

**Task 2:**

We ask you to rate your level of pleasure (from "not pleased" to "very pleased") and your state of arousal (from "very calm” to "very awake"). To do this, you have a cursor to move horizontally to express how you feel [47].


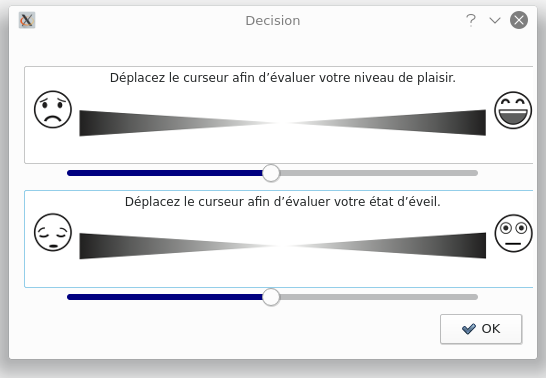


**Task 3:**

During this task, you have an endowment of €10. You are asked to make a decision regarding this endowment.

We give you the opportunity to give a part of this endowment to the ENGO (Treatments T1, T2 and T3) / HNGO (Treatment T4) you previously selected. You can choose to give any amount as an integer number between €0 and €10; you will keep the rest of the endowment to yourself.

The game will be played only once.


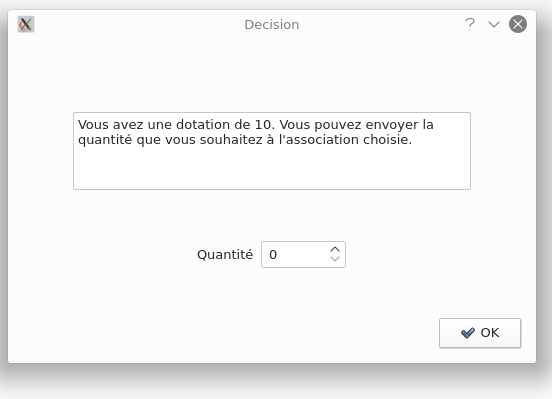


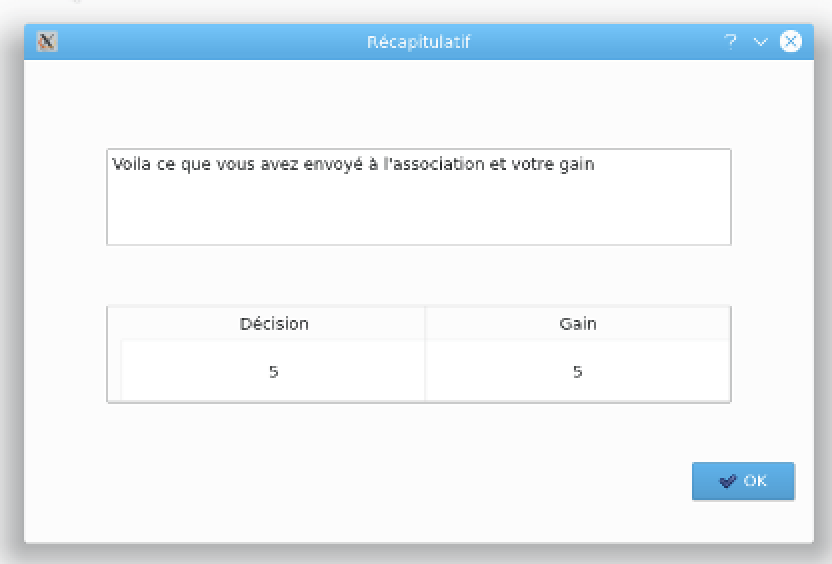


**IMPORTANT:**

The experimenters undertake on their honor to forward all the donations generated by this experiment to the chosen ENGO (Treatments T1, T2 and T3) / HNGO (Treatment T4).

**Post-experimental situation – not part of the written instructions**

At the end of the experiment, the subjects were invited one after the other to go to Room C217 to collect their earnings in private. During the payment process, subjects had the opportunity to recycle the headphone coverings in a dedicated bin.

---------------------------------------------------------------------------------------------------------------------------

**Virtual Exposure Videos**

Link to the public folder “Virtual Exposure Videos”:

<https://upvdrive.univ-montp3.fr/index.php/s/TP37yHkRbe9CAnk>

With respectively:

- The excerpt from *Walks with an architect series* from Landmark Media Inc. used in Zelenski et al. [31] entitled “Urban video”.
- The excerpt from the documentary *Wild Yellowstone* from the National Geographic Channel entitled “Nature video”.
- The excerpt of scenes from four films (*Bruce Almighty*, *Sister Act*, *What Women Want*, and *Wall-E.*) selected from Uhrig et al. [64] entitled “Positive emotions video”.

Texts of both videos, respectively the “Urban video” and the “Nature video”:

- The “Urban video”:

*“Well, when this grid is put in place, it still gives way to a path, which is the Indian path, which is the Broadway path. This Indian path certainly leads from north to south, and this diagonal it will create extremely beautiful, extremely striking events which will create heterogeneous elements in this completely homogeneous grid. And there, the Flatiron is an extremely vertical, very flat element, one of the first New York buildings, one of the first very tall buildings. And so the Flatiron built in 1903 by Burnham is, in fact, a building which is nothing other than the parcel multiplied by twenty. So, in fact it is the building, it is basically this floor area that will be multiplied.*

*So, we are here in 1903 with the Flatiron, and we're going to move on to another building which is the Woolworth building in 1913. Then we realize that the base, which is the succession of its different floors, will stop at a certain point, and above it will appear a tower. And we pass here on Equitable building, built in 1915, which will go up like that and we will multiply by sixty times the surface of the plot. This building will scare New Yorkers since the light will no longer enter the streets around it, and it will create an incredible fear among New Yorkers and it is therefore in 1916, this famous zoning laws, which will completely redesign the skyline in New York. In fact, this zoning law is extremely simple, it consists in saying that, on a given plot, the floors will be able to rise up to a certain height and from there, there are successive withdrawals, which will therefore allow in the light of patiently entering along the building to illuminate the streets to the lowest point.*

*New York is a multiplication of facts and a collection of buildings that will be represented. A bit like the Chrysler building here, a bit like the Empire state building here and so here it will be a bit like brushes in the sky that rub the underside of the stars and rub the underside of the sky.*

*Here it is, this is an extraordinary building since we are really at the origin of the elevator. This is the first Otis elevator and well, it's made for this building. And this building is still there and the elevator too. Obviously, we could not have built the buildings without having invented the elevator beforehand.*

*So, what is also extremely interesting about these buildings on the grid is that it not only seeks to constitute facades but also to create angles and to rotate the space on the angle. So, this one, the red building, we don't know if it's on Broadway or if it's on Broome Street. So, are we in front of an Italian palace? or in front of a neoclassical architecture? Or in front of a building that is neither Italian nor neoclassical? We are on Broadway! That's the specificity of Broadway, it's all of that at the same time! That's the mix, it's the place of all possibilities. What's exciting here about these buildings on Broadway is that some of them no longer hide their structure, no longer hide their skeletons. That is to say the stone disappears in favor of this structure which becomes a skin, which will give all the quality to the building.*

*Until the 1960s, it was an extremely dark, extremely opulent, very heavy building, and the metallic structure behind it, was completely hidden. Until the 1960s when, with Mies van der Rohe and the Seagram building, the structure will reappear, will express itself to give even more verticality to the building.*

*But then, if basically this plan is final, if the grid was installed extremely quickly. And from 1811, the question is to know what remains to be conquered. That is, we conquered the peninsula itself, we conquered the ground over this grid. So, the question arises: what remains to be conquered. And here I think the last object to conquer is the block. The block is located between two avenues and streets. Basically, it's a bit like, in this city that is Manhattan, where there was a whole series of cities that could be built on each of these blocks. And there is also the idea that fundamentally, the promoters, the architects will be able to dialogue with each other through interposed blogs, that is to say that basically, it's up to whoever will take the block in its entirety and build a utopia.*

*So here we are in the heart of Midtown in Manhattan, in one of the favorite places of architects The Rockefeller Center where one of the first things you have to do is dig the block. But it is not to use the block in its entirety or to use it but never to fill it completely. And consequently, he will dig the interior of the block, he will remove material, and, by removing material in this block, he will create at the very heart of the block of public space, which are the piazza, which are the aisles on a human scale. What is extraordinary here is that the buildings of the Rockefeller, of the whole of Rockefeller, come into conversation. They are supportive of each other, they are united with each other to constitute a public space below, it is a space created by the Rockefeller, which is a novelty and what will be used later in other works of architects like Mies van der Rohe when he will work on the Seagram building.*

*The Seagram building is that building, built by Mies van der Rohe on park avenue, on a gently sloping avenue, and is perhaps the paradigm of sobriety, may be the pinnacle of sobriety and elegance in New York. He is withdrawing from the alignment of the street; he is going to create a piazza here for people to sit down and have lunch between noon and 2 o'clock. he gets up very slightly, the space slides under the building. This building by Mies van der Rohe the Seagram is fascinating because deep down it's not just the street he wants to build, it's his performance space by withdrawing from the street, creating a new alignment to the even inside the block. By creating this space of representation, he also creates, perhaps, the first public spaces in New York.*

*So, one, we're on a closed peninsula. Two, we live in a final grid. Three, we are in an already determined block. Four, we have a neighborhood which constitutes the template of the building. So, what does the architect do? Well, the architect dreams.*

*New York New York of course of course, we are in the street, of course we are in motion at ground level, of course we look up. But it's also another landscape that is only perceptible beyond the twentieth floor. But when you are beyond this twentieth floor or maybe even beyond the twenty-fifth floor, there is an extraordinary thing that appears there is the invention of the new ground, the very one that we had lost vision of when we were at real ground level”.*

- The “Nature video”:

“*it's October, animals feel the approach of cold, many are leaving. Among those who remain we find animals that have already faced many winters and others for which it will be a great first. Whatever the environment in which they have taken up residence their world is about to change. In a few weeks, the water in the beaver pond will be completely frozen winter hits Yellowstone. The sudden drop in temperatures exacerbates tensions. Some animals have learned to turn the snow to their advantage. The red fox is made for winter. He weighs an average of six kilos, it is too light to really sink into the powder. So, nothing prevents him from going hunting far from his burrow but how do you find what you can't see? The solution: big ears. Its preys hide under fifty centimeters of snow, but he is able to perceive the most tenuous noise. The fox spotted a target: a meadow vole. What will he do now? If it digs, the vole will run away. So, he leaps in the air. Neither of them wants to surrender. The vole is not very big, but in winter every calorie counts. In the middle of winter, the night sky puts on a show. But this carpet of stars is often accompanied by a murderous cold. The temperature may drop below -20 degrees. Otters have already traveled 16 kilometers. And suddenly, finally this gurgling water it's the sound of life. The young otter is hungry enough to fish himself, he faced the Yellowstone winter and survived. He is ready to take his independence. Well, maybe not right away.*

*Summer is coming. With the thaw, Yellowstone completely changes its face. The flow of rivers is multiplied by eighteen; the lower falls reaches more than 183 m3 per second. Such a quantity of water inevitably makes waves. The rivers turn into a raging torrent, this is not the ideal place for a bird. But the sincle is not a bird like the others. These rapids are his hunting ground. Its short wings allow it to literally fly underwater and its dense plumage gives it perfect insulation. And this is why he goes to so much trouble, up there these little ones are safe from danger above, with an inexhaustible source of food. In Yellowstone, all parents must find a way to protect their offspring.*

*Summer is coming to an end. The earth is scorched by the sun. Even the geyser basins are starting to dry up. Mud puddles thicken. They are bubbling. But not all the water evaporates. Millions of liters seep into the ground, to the bowels of the super volcano. After about thirty years, under the effect of a colossal pressure, all this water rises to the surface and gushes out in the form of a column of superheated water and steam. Yellowstone Park has more than ten thousand geothermal phenomena making it one of the most extraordinary places in the world.”*
