# Supplementary material for: The impact of nature video exposure on pro-environmental behavior: An experimental investigation
Source: PLoS One. 2022 Nov 8;17(11):e0275806. doi: 10.1371/journal.pone.0275806 (PMC9642880; doi:10.1371/journal.pone.0275806)
Supplement: S2 File — (DOCX) [file pone.0275806.s003.docx]

**Supplementary material S2. Socio-demographic questions**

At the end of the experiment, participants were asked to respond to the following questions:

- What is your year of birth? (open-ended question).
- What is your gender? (closed-ended question: female / male).
- Are you living as a couple? (closed-ended question: yes / no).
- Are you a student? (closed-ended question: yes / no)
  - If yes, what is your field of study? (closed-ended question with an extended set of fields of study, e.g., Biology, Business School, Economics, Geography, Medicine, etc.).
  - If yes, what is your level of study? (closed-ended question: Diploma / BA – BSc / MA – MSc / PhD).
- Have you ever participated in an experiment in economics? (closed-ended question: yes / no).
